# The Tomato Cell Death Suppressor Adi3 Is Restricted to the Endosomal System in Response to the *Pseudomonas syringae* Effector Protein AvrPto

**DOI:** 10.1371/journal.pone.0110807

**Published:** 2014-10-28

**Authors:** María J. Ek-Ramos, Julian Avila, Anna C. Nelson Dittrich, Dongyin Su, Joel W. Gray, Timothy P. Devarenne

**Affiliations:** Department of Biochemistry and Biophysics, Texas A&M University, College Station, Texas, United States of America; Northeast Forestry University, China

## Abstract

The tomato (*Solanum lycopersicum*) AGC protein kinase Adi3 functions as a suppressor of cell death and was first identified as an interactor with the tomato resistance protein Pto and the *Pseudomonas syringae* effector protein AvrPto. Models predict that loss of Adi3 cell death suppression (CDS) activity during Pto/AvrPto interaction leads to the cell death associated with the resistance response initiated from this interaction. Nuclear localization is required for Adi3 CDS. Prevention of nuclear accumulation eliminates Adi3 CDS and induces cell death by localizing Adi3 to intracellular punctate membrane structures. Here we use several markers of the endomembrane system to show that the punctate membrane structures to which non-nuclear Adi3 is localized are endosomal in nature. Wild-type Adi3 also localizes in these punctate endosomal structures. This was confirmed by the use of endosomal trafficking inhibitors, which were capable of trapping wild-type Adi3 in endosomal-like structures similar to the non-nuclear Adi3. This suggests Adi3 may traffic through the cell using the endomembrane system. Additionally, Adi3 was no longer found in the nucleus but was visualized in these punctate endosomal-like membranes during the cell death induced by the Pto/AvrPto interaction. Therefore we propose that inhibiting nuclear import and constraining Adi3 to the endosomal system in response to AvrPto is a mechanism to initiate the cell death associated with resistance.

## Introduction

Programmed cell death (PCD) is part of the hypersensitive response (HR) that occurs during the resistance response of plants to pathogens [1], [2]. In tomato (*Solanum lycopersicum*), initiation of the HR and resistance to the causative agent of bacterial speck disease, *Pseudomonas syringae* pv. *tomato* (*Pst*), is brought about by the interaction of the host resistance Ser/Thr protein kinase Pto with the *Pst* effector protein AvrPto [3]. AvrPto is delivered to the host cell via the type III secretion system and localizes to the plasma membrane where it presumably interacts with Pto to initiate resistance signaling [3]–[5]. A screen for proteins involved in Pto-mediated HR induction identified the tomato Ser/Thr protein kinase Adi3 (AvrPto-dependent Pto-interacting protein 3) based on its interaction with Pto and AvrPto [6].

Adi3 has cell death suppression (CDS) activity and thus functions as a suppressor of PCD in the absence of pathogen [7], [8]. The CDS activity of Adi3 requires phosphorylation at Ser539 by the upstream kinase 3-phosphoinositide-dependent protein kinase-1 (Pdk1) and nuclear localization [7], [8]. The nuclear localization signal (NLS) of Adi3 is found in the domain known as the T-loop extension. Mutation of the NLS or deletion of the T-loop extension eliminates Adi3 nuclear localization and confines Adi3 to many intracellular punctate membranous structures, which causes cell death due to a loss of Adi3 CDS activity [8]. It is hypothesized that during pathogen challenge the interaction of Adi3 with Pto/AvrPto leads to Adi3 inactivation, a loss of Adi3 CDS, and subsequently the HR-associated PCD [7]–[9]. This raises the possibility that during Pto-mediated resistance, Adi3 is confined to these punctate membranous structures in order to bring about the HR PCD [8].

Adi3 is a member of the AGC family of protein kinases, which is a conserved family of eukaryotic Ser/Thr protein kinases regulating many basic cellular processes such as transcription, translation, cell growth, cell death, and cytoskeletal remodeling [10]. Our studies have shown that Adi3 functions remarkably similar to the mammalian AGC kinase and cell death suppressor PKB (a.k.a. Akt) [7], [8]. PKB is also phosphorylated by Pdk1 for activation, requires nuclear localization for CDS, and is found associated with endosomal vesicles similar to the punctate structures with which non-nuclear Adi3 is associated [11]. Recent studies have shown that endosomal vesicle localization plays a key role in the regulation of PKB activity and signal transduction [11], [12]. PKB has also been shown to be a target of mammalian bacterial effector proteins for manipulation of PCD during pathogenesis [13], [14].

In recent years, the endomembrane system and intracellular vesicle trafficking have been shown to play important roles in plant immunity. For example, during plant-pathogen interactions host receptors that recognize pathogen-associated molecular patterns are endocytosed upon ligand interaction, antimicrobial proteins and chemicals are exocytosed outside of the cell, and important signaling proteins are trafficked through the cell in endosomal vesicles [15]–[19]. Here we show that the non-nuclear Adi3-associated punctate structures are part of the endomembrane system and that Adi3 is restricted to the endosomal system during Pto/AvrPto-induced HR cell death. Our data suggest restriction to the endosomal system as a mechanism by which Adi3 function is subverted during the resistance response to *Pst* for induction of PCD.

## Materials and Methods

### Plasmid construction

All constructs used were previously described [8]. *Arabidopsis* endosomal markers Ara7 and RHA1 were obtained from the *Arabidopsis* Biological Resource Center and cloned into *pTEX:GFP* for N-terminal GFP translational fusions. *Arabidopsis* endosomal markers mCherry-SYP21 and mCherry-SYP61 are described in [20].

### Protoplast transient expression and endosome fractionation

Protoplasts were isolated from leaves of three-week-old PtoR or *prf-3* tomato plants and transformed with 20 µg of DNA for each construct as previously reported [7], [8]. Samples of transformed protoplasts were taken 16 hr after transformation, unless otherwise indicated, for protein expression determination, subcellular fractionation, and confocal microscopy. Endosome fractions were isolated from protoplasts expressing the indicated constructs as previously reported [21] and adapted to our system as described here. Transformed protoplasts were lysed in 100 µL of Buffer 1 (100 mM HEPES-KOH pH 7.5, 40.6% sucrose, 5 mM MgCl_2_, 2 mM β-mercaptoethanol, 10 µL/mL phosphatase inhibitors (Sigma), 10 µL/mL plant proteinase inhibitors (Sigma), 100 nM MG132, 0.2% Triton X-100, and 3 mM imidazole) by gently mixing, and 30 µL of this suspension was taken as total protein. After centrifugation at 5000 *g* for 10 min at 4°C, the supernatant (post nuclear supernatant fraction; PNF1) and the pellet (crude nuclei suspension; N1) were saved. N1 was washed with 100µL Buffer 1 three more times and each supernatant, PNF2, PNF3 and PNF4, pooled with PNF1 and used as the total post nuclear fraction (tPNF). The remaining pellet (pure nuclei-pN) was dissolved in 30 µL of Buffer 2 (10 mM MES-HCl pH 5.7, 1 M sucrose, 5 mM MgCl2, 2 mM β-mercaptoethanol, 10 µL/mL phosphatase inhibitors (Sigma), 10 µL/mL plant proteinase inhibitors (Sigma), 10 µM MG132, 1% Triton X-100), vortexed vigorously, and taken as the total nuclear fraction. The tPNF was overlaid with 500 µL Buffer 3 (same as Buffer 1 but with 35% sucrose), 500 µL of Buffer 4 (same as Buffer 1 but with 25% sucrose), and this gradient centrifuged at 40,000 *g* for 60 min at 4°C using a Beckman TL-100 ultracentrifuge and TLS-55 rotor. After centrifugation the second interphase (35%–25% sucrose) was removed, diluted 4-fold with Buffer 5 (250 mM sucrose, 10 mM triethanolamine, and 1 mM EDTA pH 7.5), and 700 µL of Buffer 6 added (17% Percoll, 10 mM triethanolamine). A self-generating percoll gradient was formed by centrifugation at 80,000 *g* for 60 min at 4°C as above. After centrifugation 10 fractions of 250 µL each were collected from top to bottom. The percoll was removed by adding 200 µL of Buffer 7 (50% sucrose) at the top if each individual fraction and centrifuging at 100,000 *g* for 2 hr at 4°C as above. 200 µL from the bottom of each sample was removed and protein precipitated using TCA/acetone. The endosome fractionation analysis was done three independent times and the presented α-GFP western blots are representative of all experiments.

### Microscopy

Transformed protoplasts were imaged 16 hr after transformation, unless otherwise indicated. For confocal microscopy, an Olympus FV1000 microscope was used as as previously described [8]. Excitation and emission wavelengths were as follows: *e*GFP, 488 nm excitation, 507 nm emission; hoechst 33342 (Sigma) and *e*BFP, 343 excitation, 460 nm emission; *Ds*RedT4, 563 nm excitation, 582 nm emission; mCherry, 587 nm excitation, 610 nm emission; FM4-64 (Invitrogen), 515 nm excitation, 620 emission; and chlorophyll autofluorescence, 470 nm excitation, 680 nm emission. All cell localization analyses with confocal microscopy were carried out at least three independent times with a minimum of 30 protoplast cells viewed each time. All confocal images shown as Z-stacks and presented images are representative of typical cell localization for all proteins seen during independent experiments. For fluorescence microscopy, a Zeiss Axioplan 2 microscope was used with an ApoTome module for optical sectioning.

### AvrPto and other treatments

For AvrPto treatment, protoplasts expressing *e*GFP-Adi3 for 16 hr were transformed with *pTEX:avrPto:FLAG* as previously described [8]. Samples were collected at 0.5, 1.5, 3 and 4 hr for confocal imaging, immunoblotting, and determination of cell viability. For other treatments, *e*GFP-Adi3 expressing protoplasts were incubated with H_2_O for 4h (no treatment), at 42°C for 30 min (heat), –20°C for 1 min and thawed at room temperature (cold), or shaken at 120 rpm at room temperature for 30 min (wounding) followed by confocal microscopy imaging. For nuclei staining, endosomal compartment identification, and wortmannin treatment, protoplasts were treated with 10 µM hoechst 33342 for 2 h, 20 µM FM4-64 for 2 h, and 33 µM wortmannin (Sigma) for 1 h, respectively, followed by confocal microscopy imaging. For colocalization studies and endosome fractionation, soybean α-1,2-mannosidase-mCherry [22], 2xFYVE-*Ds*Red, GFP-Ara7, and GFP-RHA1 were coexpressed in protoplasts for 16 hrs. All experiments were carried out a minimum of three times and images presented are representative of each experiment.

### Agrobacterium-mediated transient expression

*Agrobacterium tumefaciens* strain GV2260 was used for *Agrobacterium*-mediated transient expression in *Nb* leaves as previously reported [7]. *Adi3* constructs were expressed from pCambia1300 and *avrPto-FLAG* from pBTEX. *Nb* leaves expressing GFP-Adi3 for 24 hr were infiltrated with *A. tumefaciens* containing the *avrPto-FLAG* construct and analyzed for cell death induction 3 days later using Evans blue as previously reported [8]. Leaves were infiltrated with 20 µM FM4-64 2 hr before confocal microscopy analysis.

### Cell viability measurements

Protoplast cell viability was determined using Evans blue as previously reported [7]. All cell viability assays were carried out a minimum of three independent times.

## Results

### The Adi3^ΔT-loop^ protein is localized to the endosomal system

**Figure 1:**
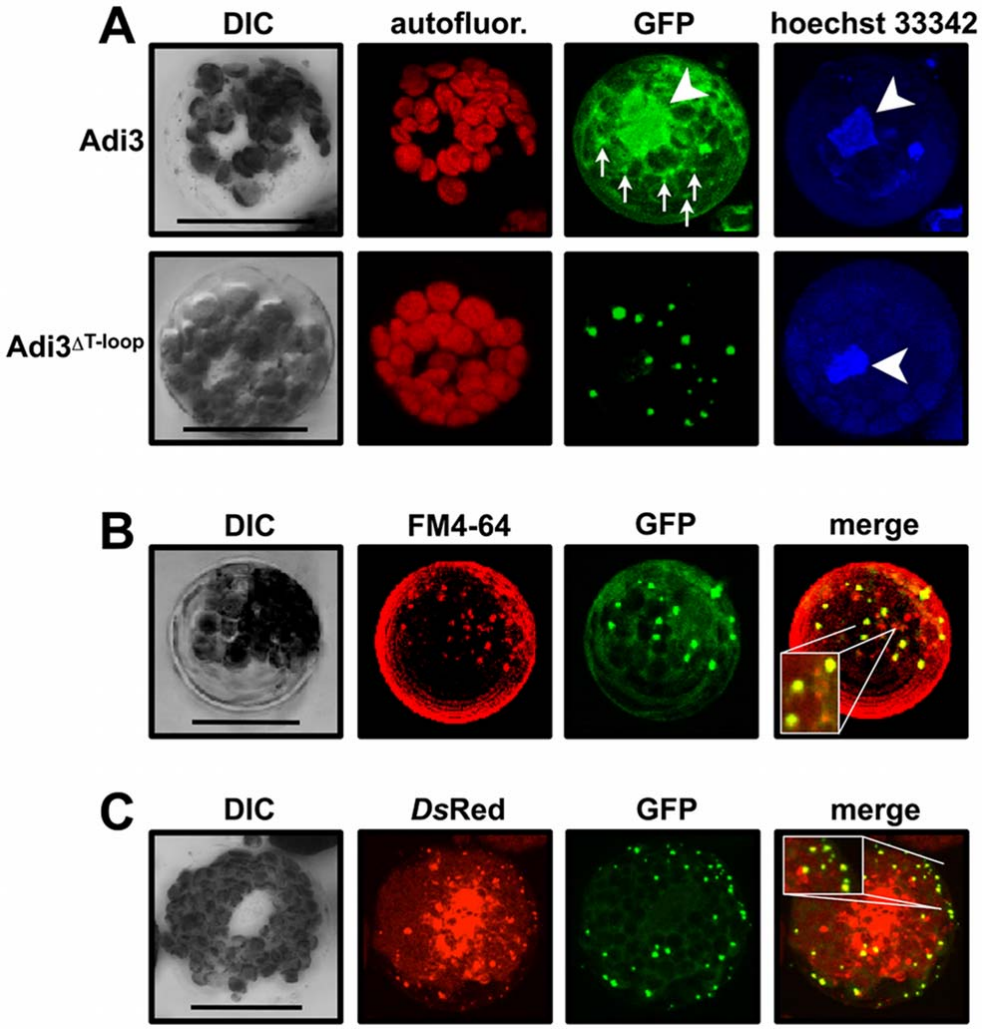
Deletion of the T-loop extension localizes Adi3 to the endomembrane system. Protoplasts of PtoR tomato plants were transformed with *GFP-Adi3* constructs, protein expressed for 16 hrs, and the GFP signal viewed by confocal microscopy. Compiled Z-axis images are shown. A, Localization of GFP-Adi3 proteins in tomato protoplasts. Protoplasts expressing the indicated GFP-Adi3 proteins were stained with Hoechst 33342 to label nuclear DNA (white arrow head). Small arrows indicate localization of punctate structures for GFP-Adi3 expression. B, GFP-Adi3^ΔT-loop^ colocalizes with the endosomal vesicle stain FM4-64. Cells expressing GFP-Adi3^ΔT-loop^ were treated with 20 µM FM4-64 for 2 hrs before visualization. Inlay shows zoom of region of interest. C, GFP-Adi3^ΔT-loop^ colocalizes with the endosomal vesicle marker 2xFYVE-*Ds*Red. *GFP-Adi3* ^*ΔT-loop*^ and *2xFYVE-DsRed* constructs were cotransformed into protoplasts and expressed for 16 hrs before visualization. Inlay shows zoom of region of interest. Bars = 20 µm.

Our previous studies have established that GFP-Adi3 cellular localization is identical in both intact leaf mesophyll cells and isolated mesophyll protoplast cells, and that protoplasts offer a better context for Adi3 cellular localization studies [8]. These studies utilized confocal microscopy and subcellular fractionation with GFP-tagged Adi3 to show that wild-type Adi3 is localized in the nucleus (Figure 1A) as well as insoluble cellular fractions, while loss of Adi3 nuclear localization by deletion of the NLS or the T-loop extension (Adi3^ΔT-loop^) localizes Adi3 to many punctate intracellular membranous cellular structures (Figure 1A) [8]. Localization of Adi3^ΔT-loop^ to these punctate structures correlates with a loss of Adi3 CDS and induction of cell death [8].

In order to determine the identity of these punctate structures to which Adi3^ΔT-loop^ is localized, tomato protoplasts coexpressing GFP-Adi3^ΔT-loop^ with protein markers for peroxisomes and mitochondria [22] were analyzed by confocal microscopy. Colocalization between these organelle markers and Adi3^ΔT-loop^ protein was not seen (Figure S1A in File S1). However, staining GFP-Adi3^ΔT-loop^ expressing protoplasts with FM4-64, a dye that is endocytosed from the plasma membrane and identifies endosomal membranes [23], [24], shows colocalization between some of the GFP and FM4-64 signals (Figure 1B, Figure S2 in File S1), suggesting Adi3^ΔT-loop^ is localized to the endosomal system.

To further confirm Adi3^ΔT-loop^ endomembrane localization, the endosomal vesicle marker 2xFYVE-*Ds*Red was used. This marker contains two FYVE domains from the mouse Hrs protein fused to *Ds*RedT4 [25]. FYVE protein domains bind phosphoinositide-3-phosphate, which is enriched in endosomal vesicles [26], and the 2xFYVE-*Ds*Red marker has been shown to localize to plant endosomal vesicles [25]. Coexpression showed GFP-Adi3^ΔT-loop^ punctate structure colocalization with some of the 2xFYVE-*Ds*Red labeled endosomal vesicles (Figure 1C, Figure S3 in File S1). In time-lapse imaging, these colocalizing signals had synchronous movement (Movie S1; 5 min movie, 10 frames, 30 seconds each). Quantitation of GFP-Adi3^ΔT-loop^/FM4-64 and GFP-Adi3^ΔT-loop^/2xFYVE-*Ds*Red colocalization using 10 protoplasts from 5 independent experiments showed 61.4±5.8% and 93.4±1.2% colocalization, respectively (Figure S1B in File S1).

We also examined colocalization with the Golgi apparatus, another compartment of the endosomal system. Coexpression of GFP-Adi3^ΔT-loop^ with the targeting sequence of the Golgi stack localized soybean α-1,2-mannosidase fused to mCherry [22] did not show colocalization (Figure S1A in File S1). However, many GFP-Adi3^ΔT-loop^ punctate structures were seen in close proximity to the mCherry signal (Figure S1A in File S1) suggesting some GFP-Adi3^ΔT-loop^ may be closely associated with the Golgi stacks. Taken together these data suggest the GFP-Adi3^ΔT-loop^ protein is localized to the endomembrane system. The finding that not all GFP-Adi3^ΔT-loop^ signals colocalize with the markers could be indicative of dynamic GFP-Adi3^ΔT-loop^ localization to a mixture of endosomal compartments.

### Wild-type Adi3 is also localized to the endosomal system

**Figure 2:**
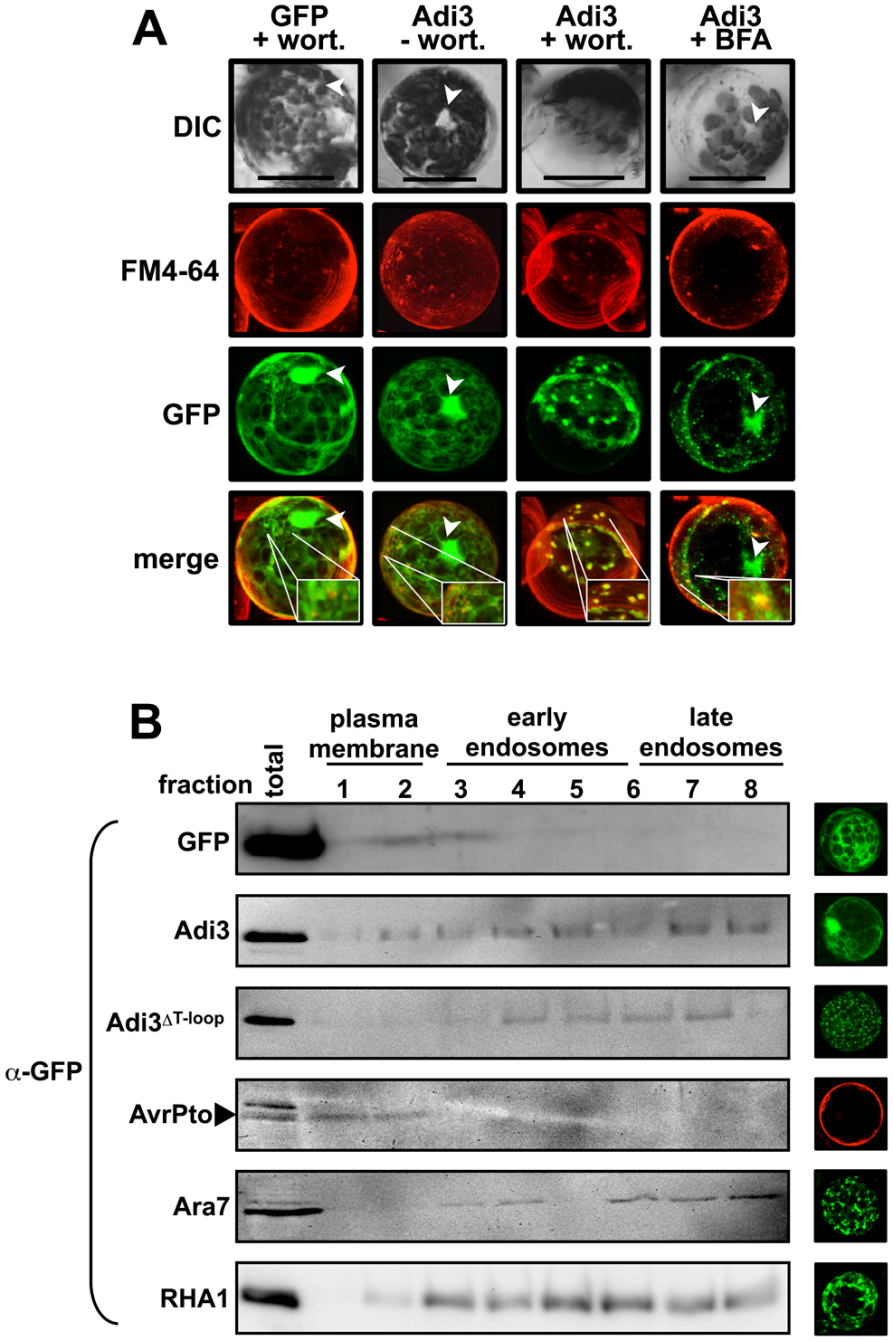
Wild-type Adi3 can be found in endosomal vesicles. A, Wortmannin and BFA treatment restricts Adi3 to endosomal vesicles. Protoplasts expressing GFP-Adi3 or GFP for 16 hrs were treated with 20 µM FM4-64 for 1 hr, treated with 33 µM wortmannin or 25 µM BFA for 1 hr, and visualized. Compiled Z-axis images are shown. Inlays show zoom of region of interest. Bars = 20 µm. B, Confirmation of GFP-Adi3 protein localization to the endosomal system. The indicated GFP-Adi3 proteins were expressed in protoplasts for 16 hrs and subjected to a sucrose/percoll double gradient and fractionation followed by α-GFP western blot on each fraction. AvrPto-BFP expressed in *prf-3* protoplasts for 1 hr was used as plasma membrane marker. *Arabidopsis* GFP-Ara7 and GFP-Rha1 were used as endosomal markers. Confocal images show representative localization for each construct. AvrPto-BFP false colored red.

GFP-Adi3 protein has been found only in nuclear and insoluble cellular fractions by subcellular fractionation, but not the soluble fraction even though GFP-Adi3 signal is observed in what appears to be the cytoplasm by microscopy (Figure 1A) [8]. This apparent cytoplasmic GFP-Adi3 signal is not due to free GFP as we have shown that GFP-Adi3 protein does not degrade into free GFP and Adi3 [8]. This would suggest the apparent GFP-Adi3 cytoplasmic signal is actually localized to intracellular membranes, possibly endosomal membranes. In fact, GFP-Adi3 can be seen localized to several punctate structures similar to GFP-Adi3^ΔT-loop^ (small arrows in Figure 1A). In order to test GFP-Adi3 localization to the endosomal system, GFP-Adi3 expressing protoplasts were treated with the phosphatidylinositol-3 kinase inhibitor wortmannin, which inhibits endocytosis at the plasma membrane as well as fusion of recycling endosomes to the TGN [27]. Thus, endosomal resident proteins accumulate at the plasma membrane and/or in endosomal vesicles. Wortmannin treatment showed a loss of nuclear GFP-Adi3, a reduction in apparent cytoplasmic GFP-Adi3, and the appearance of GFP-Adi3 in punctate structures, some of which colocalized with FM4-64 (Figure 2A, Figure S4 in File S1). GFP-Adi3 expressing protoplasts were also treated with brefeldin A (BFA), another endosomal trafficking inhibitor which inhibits ARF-GTPases involved in endosomal trafficking [28]. Thus, endosomally localized proteins are trapped in the endosomal system [28]. BFA treatment showed an accumulation of GFP-Adi3 in punctate structures, some of which colocalize with FM4-64 (Figure 2A, Figure S5 in File S1). However, BFA was not capable of completely eliminating GFP-Adi3 nuclear localization (Figure 2A). This may be due to the variable nature of BFA effects, which have been reported to vary depending on the concentration used and the plant and tissue studied. For example, Arabidopsis protoplasts are sensitive to BFA, while maize protoplasts are not [29], [30], and different sizes of tomato hypocotyl protoplasts have shown different sensitivity to BFA for the formation of punctate structures [31]. This could explain why our tomato protoplasts may not be completely responsive to BFA.

These data with endosomal trafficking inhibitors suggest Adi3 is a resident protein of the endomembrane system. Taken together with our previous studies [8] it appears that part of the Adi3 protein population is found in the endosomal system as well as the nucleus, and interruption of nuclear localization (Adi3^ΔT-loop^ or Adi3 NLS mutant) restricts Adi3 to the endomembrane system eliminating its CDS activity.

### Confirmation of Adi3 endosomal localization

The endosomal localization of GFP-Adi3 and the GFP-Adi3^ΔT-loop^ proteins was confirmed using a two-step fractionation protocol utilizing sucrose and percoll gradients to isolate plasma membrane and early endosome (EE) and late endosome (EE) cellular fractions [21]. It should be noted that using this qualitative protocol plasma membrane (lighter), EE (medium), and LE proteins (heavier) are separated into fractions that will overlap with each other. AvrPto-BFP was used as a plasma membrane marker [32] and GFP fusions of the *Arabidopsis* Rab GTPases Ara7 and Rha1 were used as endosomal markers [33]–[35]. The results indicate that Ara7 and Rha1 distribute mainly in heavier fractions correlating to EE and LE (Figure 2B). Interestingly, wild-type Adi3 was found in all fractions, while Adi3^ΔT-loop^ protein was found mainly in the EE and LE fractions (Figure 2B). These data support our contention that wild-type Adi3 is a resident protein of the endosomal system and deletion of the Adi3 T-loop extension (prevention of nuclear localization) restricts Adi3 to the endosomal system.

In an attempt to further characterize the endosomal compartment to which the Adi3^ΔT-loop^ protein is localized we coexpressed this protein with mCherry fusions to two SNARE complex proteins involved in vesicle trafficking/fusion; SYP61 and SYP21, which have been shown to be TGN/EE and prevacuolar compartment/LE localized, respectively [36]. While localization of both mCherry-SYP61 and mCherry-SYP21 was similar to what has been previously seen (punctate cellular structures; [20], [36]), there was no colocalization with Adi3^ΔT-loop^ punctate cellular structures (Figures S6 and S7 in File S1). This suggests that the Adi3^ΔT-loop^ protein is not localized to the same EE or LE compartments as SYP61 or SYP21, and it will be important research for the future to determine the endosomal compartment to which the Adi3^ΔT-loop^ protein is localized.

The *Pseudomonas* effector protein AvrPto induces endosomal localization of Adi3.

**Figure 3:**
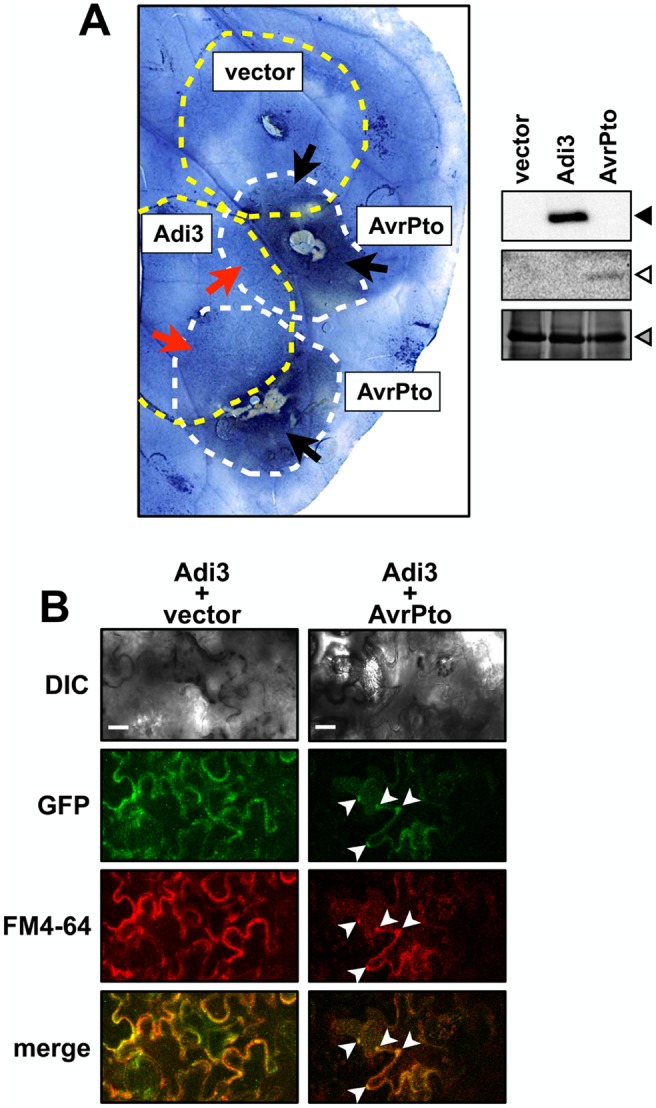
AvrPto induces Adi3 endosomal localization. A, GFP-Adi3 suppression of AvrPto-induced cell death in *Nb* leaves. GFP-Adi3 or empty vector (yellow dashed circles) were expressed by *Agrobacterium*-mediated transient expression for 24 hrs followed by expression of AvrPto-FLAG (white dashed circles) so that the two expression zones overlapped. After 72 hrs, leaves were stained with Evans blue, de-pigmented, and photographed. The image presented is representative of 3 independent leaves. Red arrows, zones of Adi3 suppression of AvrPto-induced cell death; black arrows, zones of AvrPto-induced cell death. B, Confocal images of GFP-Adi3 localization in *Nb* leaf epidermal cells in the presence of vector only (left column) or AvrPto (right column) from regions indicated by red arrows in A. 20 µM FM4-64 was infiltrated and incubated for 2 hrs before imaging. White arrowheads, GFP-Adi3/FM4-64 colocalization. Bars = 20 µm.

Since endosomal Adi3 induces cell death due to a loss of CDS [8], the Pto/AvrPto interaction leads to HR cell death [3], and Adi3 was originally isolated based on interaction with Pto and AvrPto [6], we examined if Adi3 is endosomally localized during the HR cell death induced by AvrPto. GFP-Adi3 and AvrPto-FLAG were expressed by *Agrobacterium*-mediated transient expression in overlapping zones in leaves of *Nicotiana benthamiana* (*Nb*), which contains three *Pto* homologs and produces HR cell death in response to AvrPto [37]. After 72 hrs of expression, leaves were stained with Evans blue, which stains dead cells [38], and depigmented to visualize Evans blue-stained cell death zones. In leaf regions where only AvrPto-FLAG was expressed, strong cell death staining was seen (Figure 3A, black arrows). However, in leaf regions where both AvrPto-FLAG and GFP-Adi3 were expressed, GFP-Adi3 showed CDS activity against AvrPto-FLAG (Figure 3A, red arrows). It should be noted that in the AvrPto-FLAG/GFP-Adi3 coexpression regions there are some Evans blue-stained dead cell regions indicating GFP-Adi3 is not completely suppressing AvrPto-FLAG-induced cell death (Figure 3A). This matches our previous studies using tomato protoplasts that showed GFP-Adi3 can suppress AvrPto-FLAG-induced cell death, but not completely [8]. Duplicate leaves were stained with FM4-64 and the GFP and FM4-64 signals viewed by confocal microscopy in epidermal cells. In the regions of only GFP-Adi3 expression, the GFP and FM4-64 signals were seen throughout the cell (Figure 3B, left column). However, in regions where GFP-Adi3 and AvrPto were expressed and some level of cell death was occurring, GFP-Adi3 cytoplasmic localization was reduced and colocalized with punctate structures stained by FM4-64 (Figure 3B, right column, white arrows). It should be noted that imaging of leaves expressing GFP-Adi3 and AvrPto required increased laser power in order to detect the GFP signal due to the cell death that is occurring. This gives a high background and a similar localization pattern as that of the controls. Thus, the identity of the punctuate structures in this situation should be taken with caution.

**Figure 4:**
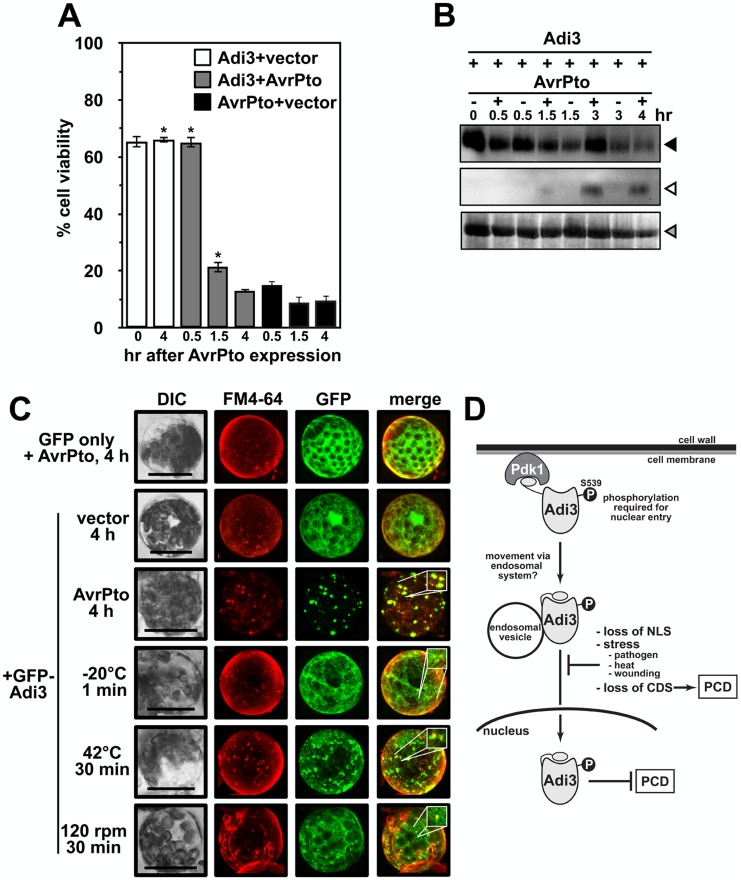
Stress induced endosomal localization of Adi3. A, AvrPto-induced cell death in PtoR protoplasts. GFP-Adi3 expressing protoplasts were transformed with an *avrPto-FLAG* construct and cell death monitored for 4 hrs with Evans blue. B, α-GFP western blot for GFP-Adi3 and α-FLAG western for AvrPto-FLAG expression in PtoR tomato protoplasts from A. Filled triangle, GFP-Adi3; open triangle, AvrPto-FLAG; gray triangle, RuBisCo loading control. C, GFP-Adi3 localization in response to stresses. PtoR protoplasts expressing GFP-Adi3 for 16 hrs were exposed to pathogen stress (AvrPto-FLAG expression), cold stress (−20°C), heat stress (42°C) and wounding (shaking at 120 rpm), FM4-64 (20 µM) stained for 2 hrs, and visualized by confocal microscopy. Inlays show zoom of region of interest. Bar = 20 µm. D, A model for Adi3 function in CDS and loss of Adi3 CDS in response to stress. See text for details.

For this reason similar assays were carried out in protoplasts. As stated above, our previous studies have shown that GFP-Adi3 behaves the same in leaf tissue and mesophyll protoplasts, and localization studies are more precise in protoplast cells, especially those undergoing cell death because the higher laser power needed for detecting the GFP signal does not cause high background [8]. As with all our protoplast assays, PtoR tomato plants containing the endogenous *Pto* gene were used for protoplast isolation. GFP-Adi3 expressing protoplasts were transformed with *avrPto-FLAG* and cell viability determined over a 4 hr AvrPto-FLAG expression period (Figure 4A). In identical assays, we have shown that GFP-Adi3 is capable of suppressing AvrPto-FLAG-induced cell death up to 1.5 hrs after AvrPto-FLAG expression, but not after 4 hrs of AvrPto-FLAG expression [8]. Both AvrPto-FLAG and GFP-Adi3 could be detected by western blot over the entire 4 hr expression time course (Figure 4B). Thus, there are some cells expressing both proteins and undergoing cell death at this 4 hr time point. This is supported by the strong AvrPto-FLAG-induced cell death seen at 4 hrs with or without Adi3 (Figure 4A). Localization of GFP-Adi3 during cell death was analyzed using confocal microscopy and showed GFP-Adi3 strongly localized to FM4-64 stained endosomal vesicles in response to AvrPto-FLAG at the 4 hr time point (Figure 4C) that is very similar to the localization of Adi3^ΔT-loop^ (Figure 1A). Additionally, GFP-Adi3 was not seen associated with the nucleus in response to AvrPto-FLAG (Figure 4C). To support that Adi3 endosomal localization is due to Pto/AvrPto-induced cell death, we carried out the same assay in protoplasts from *prf-3* plants, which contain a loss-of-function deletion in the *Prf* gene [39]. Prf is required for operative resistance signaling during the Pto/AvrPto interaction and AvrPto does not induce HR cell death in *prf-3* plants [3], [8]. We saw that Adi3 cell localization was unaffected by AvrPto expression in *prf-3* protoplasts (Figure S8 in File S1). These data indicate there is a correlation between endosomal localization of GFP-Adi3 and cell death induced by AvrPto during Pto-mediated resistance.

### Other cell death inducing stresses localize Adi3 to the endosomal compartment

In plants, PCD is also known to be involved in the abiotic stresses heat [40], [41] and cold shock [42], [43], as well as wounding [44]. Changes in Adi3 cellular localization in response to these stresses was also analyzed to determine if Adi3 endosomal localization could be a general mechanism for PCD induction during stress. GFP-Adi3 expressing tomato protoplasts were stained with FM4-64, treated with heat, cold, or wounding, and viewed by confocal microscopy. All treatments were capable of eliminating Adi3 nuclear accumulation and localizing Adi3 to endosomal-like structures that colocalized with FM4-64 stained endosomes (Figure 4C). However, it should be noted that the endosomal-like structure localization for these treatments is not as strong as that for the response to AvrPto (Figure 4C). This is most likely due to the strength of PCD induction, which is very strong and rapid in response to AvrPto.

## Discussion

### Adi3 is an endosomal resident protein

We present several lines of evidence that Adi3 is found within the endosomal system during the life cycle of the protein. The endosomal trafficking inhibitor wortmannin causes an accumulation of GFP-Adi3 in endosomal-like vesicles (Figure 2A) consistent with wortmannin function [27]. Furthermore, subcellular fractionation showed GFP-Adi3 is localized in endomembrane fractions (Figure 2B). Also, prevention of Adi3 nuclear accumulation by deletion of the Adi3 T-loop extension or NLS mutation caused retention of Adi3 in endosomal-like vesicles (Figure 1A, B, C) [8]. Taken together these data suggest that Adi3 utilizes the endosomal system for cellular trafficking.

There are relatively few reports of endosomal vesicle localization for other AGC kinases. In plants, the PHOT1 blue light receptor is associated with endocytic vesicles upon activation [45] and the auxin transport regulator PID is localized to punctate structures upon stimulation that could be endosomal in nature [46]. Two mammalian AGC kinases, CISK and PKB, both of which function as signaling kinases to regulate anti-apoptotic pathways in a manner similar to Adi3, are localized to endosomal vesicles [47]–[50]. Specifically, PKB is associated with endosomal vesicles in response to growth factor and hormone treatment [11], [12], [51], [52]. These studies and our results may indicate that many mammalian and plant AGC kinases are localized to endosomal vesicles possibly for signaling, cellular movement and/or degradation. In fact, it has been proposed that many plant AGC kinases are localized to endosomal vesicles upon perception of external stimuli [53].

From our data we propose a model for Adi3 movement to the nucleus (Figure 4D). Since wild-type Adi3 is found in the nucleus [8] and in the endosomal system (Figure 2A, 2B), and non-nuclear Adi3 (T-loop deletion or NLS mutation) is found in endosomal vesicles (Figure 1A) [8], our data suggest Adi3 may traffic to the nucleus through the endomembrane system. This is similar to the epidermal growth factor receptor (EGFR) ErbB2, which utilizes endosomal vesicles for nuclear translocation upon endocytosis in response to growth factor binding [54]. Mutation of the ErbB2 NLS no longer allows nuclear entry, but ErbB2 is still endocytosed into endosomal vesicles [54].

It is important to note that nuclear transport *via* the endosomal system as we propose (Figure 4D) is not a new phenomenon. However, it is an under studied field. Several endocytosed membrane receptor kinases such as EGFRs and proteins associated with the cytoplasmic face of endosomal vesicles utilize endosomal trafficking for nuclear entry [55]–[59]. However, the exact mechanism(s) is unknown and several routes have been proposed. The first mechanism involves retrograde vesicle movement of the proteins through the Golgi and ER, which would allow the cytoplasmic NLS to interact with the nuclear import machinery as it travels through the ER to the nucleus [55], [59], [60]. A second mechanism entails the proteins being released from the vesicle into the cytoplasm for interaction with the nuclear import machinery [55], [59], [61]. In a third mechanism, the cytoplasm exposed NLS of an endosomal vesicle-associated protein is recognized by importin-β in order to direct the vesicle to the nuclear import machinery for nuclear import of the target protein [54], [59], [62]. Also in support of our model is the finding that PKB movement to the nucleus is associated with vesicles since the Rho GTPase, RhoB, which localizes to endosomes and functions in vesicle trafficking, is required for PKB nuclear movement [49]. Our model (Figure 4D) also depicts Adi3 associated with the cytoplasmic face of endosomal vesicles since Adi3 is an intracellular protein, has no obvious membrane spanning region, and exposure to the cytoplasm would be required for Adi3 interaction with the nuclear import machinery.

Another important question is how does Adi3 associate with the endosomal system? Initiation of Adi3 endosomal localization may be originate by Pdk1 phosphorylation since this event occurs at the plasma membrane and is required for Adi3 nuclear entry [7], [8]. Phosphorylation has been shown to be required for association of some proteins to endocytic machinery in animal systems and has been suggested to be required for plants systems [63]. Another alternative for Adi3 endosomal localization is ubiquitination, a posttranslational modification that not only targets proteins for degradation, but can also act as a signal for protein endosomal localization [64]–[66]. While we have shown Adi3 ubiquitination leads to its degradation [67], ubiquitination may also be a signal for Adi3 endosomal association. This would be similar to what has been reported for the *Arabidopsis* flagellin receptor FLS2, which is ubiquitinated leading to proteasomal degradation [68], and has been suggested to be required for FLS2 endosomal localization [69].

### Endosomal localization as a mechanism for loss of Adi3 CDS during stress

Given the correlation between loss of CDS for endosomal Adi3 [8] and AvrPto-induced endosomal localization of Adi3 in the presence of a functional Pto pathway (Figure 4C), our data support a model in which Adi3 endosomal retention is a mechanism to prevent Adi3 nuclear import for inactivation of its CDS activity in response to *Pst* (Figure 4D). This would bring about HR-associated PCD. This is of significance as little is known about how cell death is regulated in plants, and recently endosomal localization of host proteins has emerged as an important process in plant resistance to pathogens. FLS2 and the tomato receptor Eix2, which recognizes the fungal EIX protein, are known to be endocytosed upon ligand interaction to initiate PAMP-triggered immunity (PTI; basal defense) responses [69]–[71]. FLS2 may even initiate signaling from endosomes [15], [18]. HR-based resistance initiated from the interaction of host resistance proteins with pathogen effector proteins known as effector-triggered immunity (ETI), such as Pto/AvrPto, has also been suggested to involve endocytosis [15]. In support of this, a broad range of resistance proteins have been shown to interact with the endosomally localized CRT1 ATPase/kinase from *Arabidopsis*, which is required for early signaling events in the resistance response against several pathogens [72]. Our finding that Adi3 is retained in endosomes in response to AvrPto further supports endosomal vesicle trafficking as having important roles in host resistance mechanisms.

One potential mechanism for Adi3 endosomal retention during PCD induction is the interaction with AvrPto. Even though AvrPto has been shown to localize to the plasma membrane [4], localization of AvrPto to the endosomal system has also been suggested based on its interaction with RabE family GTPases involved in vesicle trafficking [6], [73]. Additionally, a fraction of cellular AvrPto can be found associated with intracellular membranes [4]. Thus, it is possible that endosomal AvrPto may prevent Adi3 nuclear accumulation and retain Adi3 in endosomal vesicles for loss of CDS (Figure 4D). Additionally, we have found that Pto is capable of phosphorylating Adi3 [7], which may also contribute to Adi3 endosomal retention. However, we have not been successful in identifying the Pto phosphorylation site on Adi3 that would facilitate such studies. Our results also show Adi3 localization to the endosomal system during cold, heat, and wounding stresses. Thus, the restriction of Adi3 to endosomes may be a general mechanism plants employ to regulate cell death under different environmental stresses. In the future, it will be important to address how Adi3 functions in the nucleus for CDS and further support nuclear trafficking on endosomes.

## Supporting Information

File S1 **This file contains Figure S1–Figure S8. Figure S1**, Coexpression of GFP-Adi3^ΔT-loop^ with cellular organelle markers. A, GFP-Adi3^ΔT-loop^ was expressed in protoplast cells with *m*Cherry translational fusions to targeting sequences for the indicated organelle as described in Nelson et al. (2007). Proteins were coexpressed for 16 hrs and viewed by confocal microscopy. Bar = 20 µm. B, Percentage of GFP-Adi3^ΔT-loop^ colocalization with FM4-64 and 2xFYVE-*Ds*Red labeled endosomes. Error bars are standard error. **Figure S2**, GFP-Adi3^ΔT-loop^/FM4-64 colocalization. Images around central image are confocal microscopy close-up images of GFP/FM4-64 colocalization in individual Z-axis slices. In these images; top, GFP signal; middle, FM4-64 signal; bottom, merge. Central image shows combination of all Z-axis images for the merged GFP and FM4-64 signals. **Figure S3**, GFP-Adi3^ΔT-loop^/FYVE-*Ds*Red colocalization. Images around central image are confocal microscopy close-up images of GFP/*Ds*Red colocalization in individual Z-axis slices. In these images; top, GFP signal; middle, *Ds*Red signal; bottom, merge. Central image shows combination of all Z-axis images for the merged GFP and *Ds*Red signals. **Figure S4**, GFP-Adi3/FM4-64 colocalization after wortmannin treatment. Images around central image are confocal microscopy close-up images of GFP/FM4-64 colocalization in individual Z-axis slices. In these images; top, GFP signal; middle, FM4-64 signal; bottom, merge. Central image shows combination of all Z-axis images for the merged GFP and FM4-64 signals. **Figure S5**, GFP-Adi3/FM4-64 colocalization after brefeldin A treatment. Images around central image are confocal microscopy close-up images of GFP/FM4-64 colocalization in individual Z-axis slices. In these images; top, GFP signal; middle, FM4-64 signal; bottom, merge. Central image shows combination of all Z-axis images for the merged GFP and FM4-64 signals. **Figure S6**, GFP-Adi3^ΔT-loop^/*m*Cherry-SYP61 colocalization. Fluorescent microscope images of six independent protoplasts showing localization of GFP-Adi3^ΔT-loop^ and *m*Cherry-SYP21. Constructs were cotransformed into protoplasts, incubated for 16 hrs, and 50 µM dexamethasone added to induce *m*Cherry-SYP21 expression. Cells were viewed by fluorescent microscopy XX hrs after dexamethasone treatment. The *m*Cherry-SYP21 construct was described previously (Gu and Innes, 2011, Plant Physiol. 155:1827). Bar = 20 µm. **Figure S7**, GFP-Adi3^ΔT-loop^/*m*Cherry-SYP21 colocalization. Fluorescent microscope images of five independent protoplasts showing localization of GFP-Adi3^ΔT-loop^ and *m*Cherry-SYP21. Constructs were cotransformed into protoplasts, incubated for 16 hrs, and 50 µM dexamethasone added to induce *m*Cherry-SYP21 expression. Cells were viewed by fluorescent microscopy XX hrs after dexamethasone treatment. The *m*Cherry-SYP21 construct was described previously (Gu and Innes, 2011, Plant Physiol. 155:1827). Bar = 20 µm. **Figure S8**, GFP-Adi3 localization in the presence of AvrPto in *prf-3* tomato protoplasts. *prf-3* protoplasts expressing GFP-Adi3 for 16 hrs were transformed with an *AvrPto-mCherry* construct, the protein allowed to express for an additional 4 hrs, and the GFP and mCherry signals viewed by confocal microscopy. GFP and mCherry were viewed in the same cell. Arrowhead, nucleus; bar, 20 µm.
(PDF)

Movie S1 **Time-lapse imaging showing colocalizing signals for GFP-Adi3ΔT-loop punctate structures with 2xFYVE-DsRed; 5** **min movie, 10 frames, 30** **seconds each.**
(MP4)
