# Supplementary material for: The Tomato Cell Death Suppressor Adi3 Is Restricted to the Endosomal System in Response to the Pseudomonas syringae Effector Protein AvrPto
Source: PLoS One. 2014 Oct 28;9(10):e110807. doi: 10.1371/journal.pone.0110807 (PMC4211712; doi:10.1371/journal.pone.0110807)
Supplement: File S1 — This file contains Figure S1–Figure S8. Figure S1, Coexpression of GFP-Adi3ΔT-loop with cellular organelle markers. A, GFP-Adi3ΔT-loop was expressed in protoplast cells with mCherry translational fusions to targeting sequences for the indicated organelle as described in Nelson et al. (2007). Proteins were coexpressed for 16 hrs and viewed by confocal microscopy. Bar = 20 µm. B, Percentage of GFP-Adi3ΔT-loop colocalization with FM4-64 and 2xFYVE-DsRed labeled endosomes. Error bars are standard error. Figure S2, GFP-Adi3ΔT-loop/FM4-64 colocalization. Images around central image are confocal microscopy close-up images of GFP/FM4-64 colocalization in individual Z-axis slices. In these images; top, GFP signal; middle, FM4-64 signal; bottom, merge. Central image shows combination of all Z-axis images for the merged GFP and FM4-64 signals. Figure S3, GFP-Adi3ΔT-loop/FYVE-DsRed colocalization. Images around central image are confocal microscopy close-up images of GFP/DsRed colocalization in individual Z-axis slices. In these images; top, GFP signal; middle, DsRed signal; bottom, merge. Central image shows combination of all Z-axis images for the merged GFP and DsRed signals. Figure S4, GFP-Adi3/FM4-64 colocalization after wortmannin treatment. Images around central image are confocal microscopy close-up images of GFP/FM4-64 colocalization in individual Z-axis slices. In these images; top, GFP signal; middle, FM4-64 signal; bottom, merge. Central image shows combination of all Z-axis images for the merged GFP and FM4-64 signals. Figure S5, GFP-Adi3/FM4-64 colocalization after brefeldin A treatment. Images around central image are confocal microscopy close-up images of GFP/FM4-64 colocalization in individual Z-axis slices. In these images; top, GFP signal; middle, FM4-64 signal; bottom, merge. Central image shows combination of all Z-axis images for the merged GFP and FM4-64 signals. Figure S6, GFP-Adi3ΔT-loop/mCherry-SYP61 colocalization. Fluorescent microscope images of six [file pone.0110807.s001.pdf]

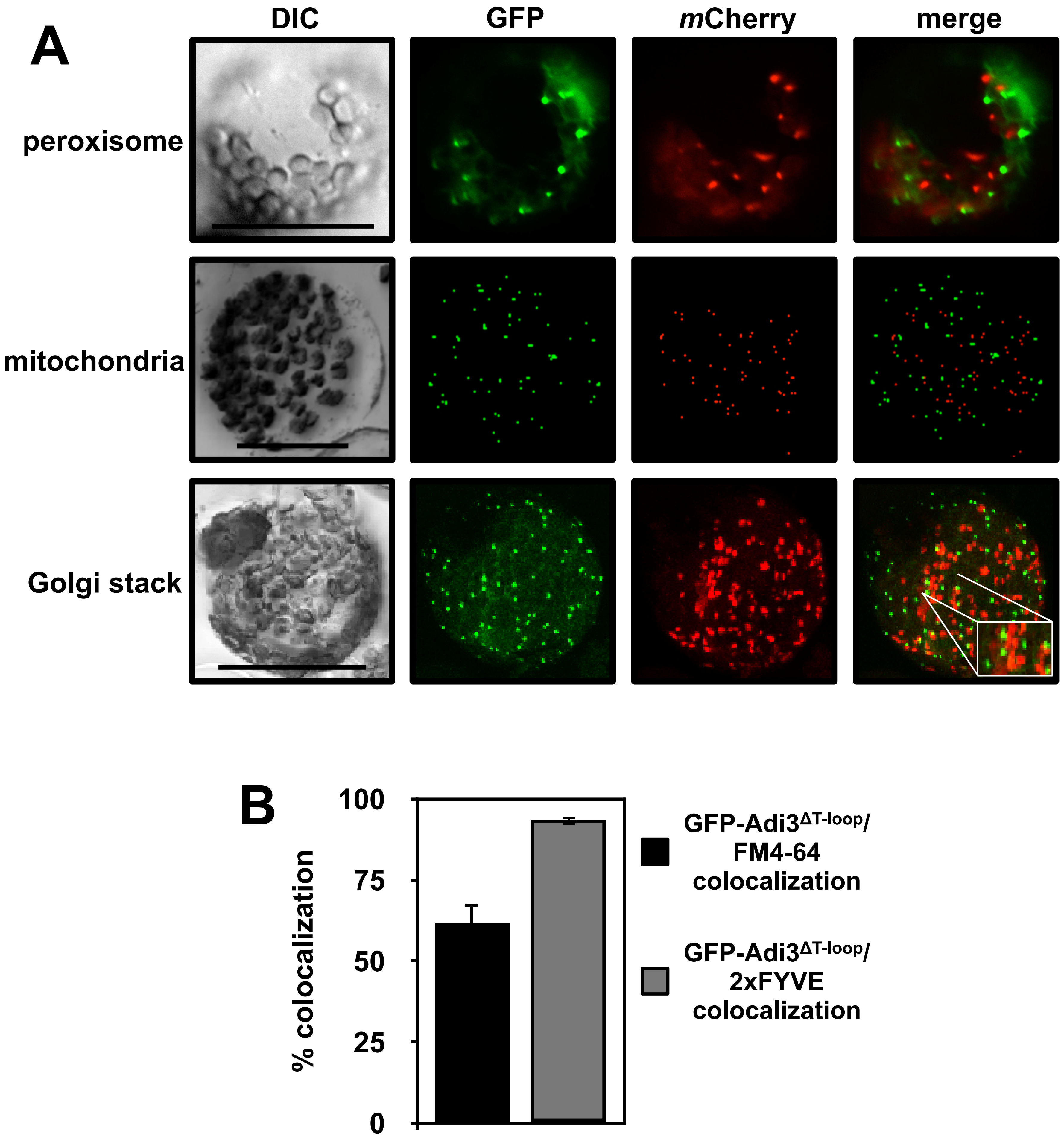

Figure S1. Coexpression of GFP-Adi3$^{\Delta\text{T-loop}}$ with cellular organelle markers. A, GFP-Adi3$^{\Delta\text{T-loop}}$ was expressed in protoplast cells with *m*Cherry translational fusions to targeting sequences for the indicated organelle as described in Nelson et al. (2007). Proteins were coexpressed for 16 hrs and viewed by confocal microscopy. Bar = 20 µm. B, Percentage of GFP-Adi3$^{\Delta\text{T-loop}}$ colocalization with FM4-64 and 2xFYVE-*Ds*Red labeled endosomes. Error bars are standard error.

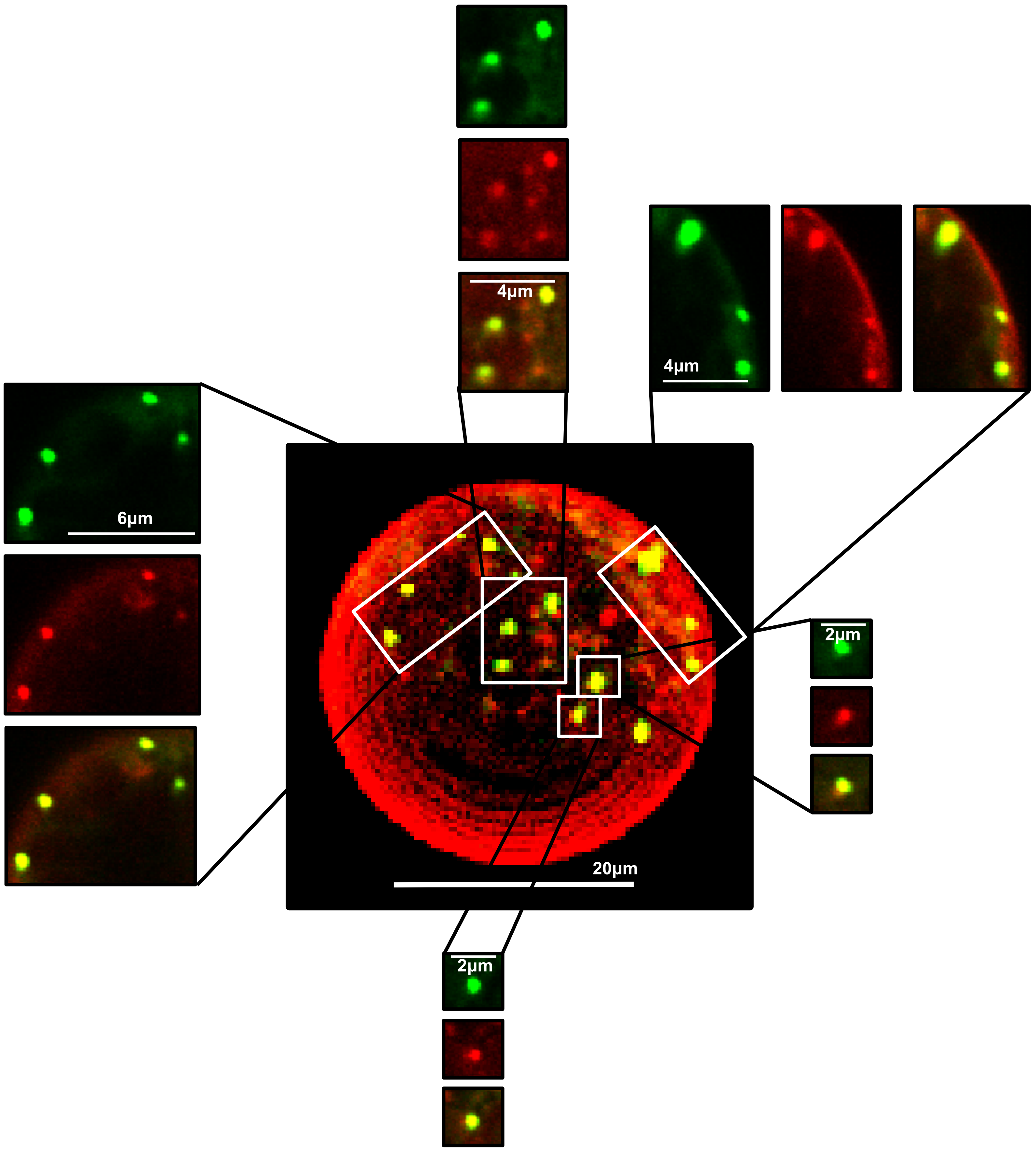

Figure S2. GFP-Adi3$^{\Delta T\text{-loop}}$/FM4-64 colocalization. Images around central image are confocal microscopy close-up images of GFP/FM4-64 colocalization in individual Z-axis slices. In these images; top, GFP signal; middle, FM4-64 signal; bottom, merge. Central image shows combination of all Z-axis images for the merged GFP and FM4-64 signals.

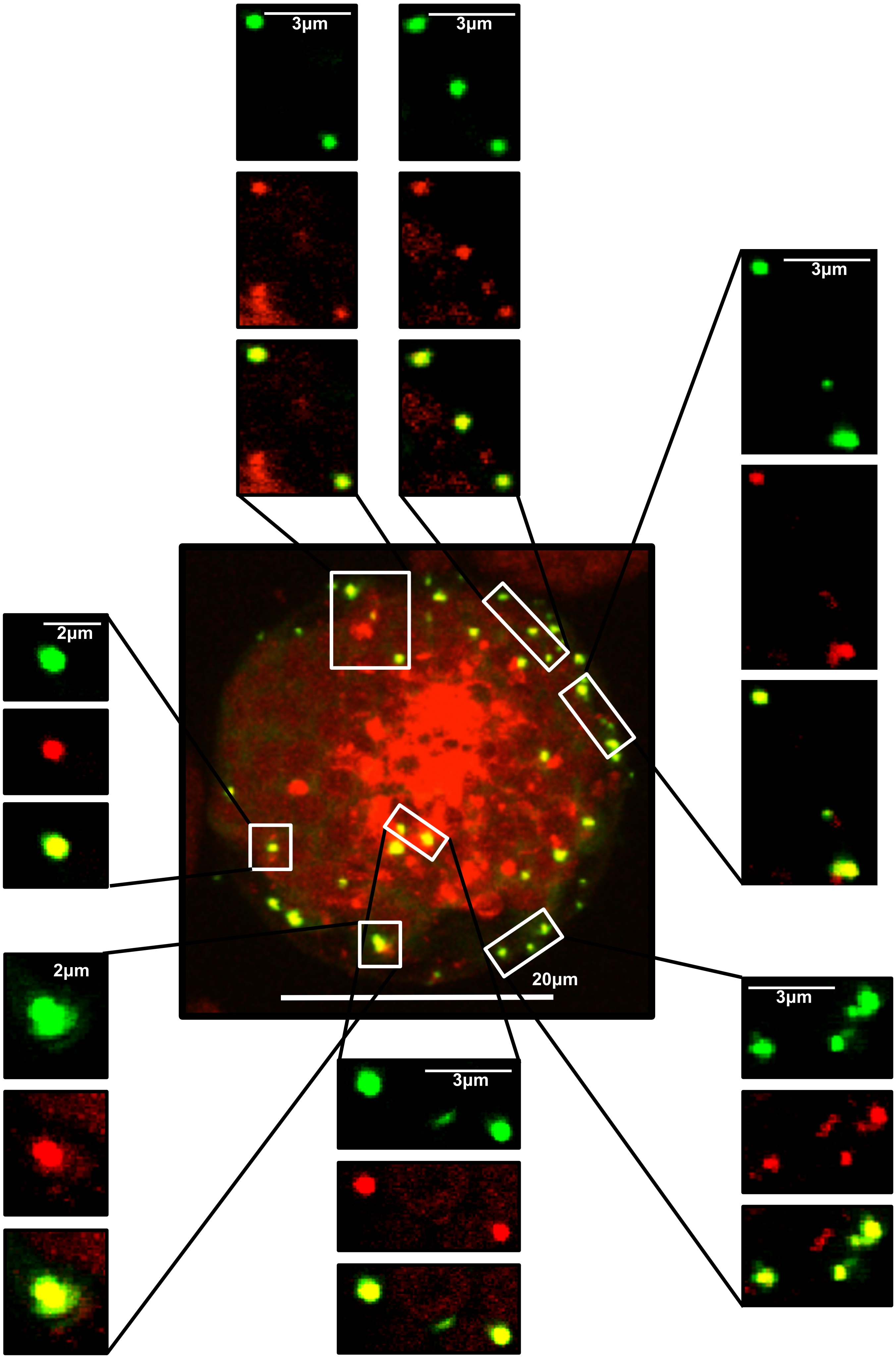

Figure S3. GFP-Adi3$^{\Delta T\text{-loop}}$/FYVE-*Ds*Red colocalization. Images around central image are confocal microscopy close-up images of GFP/*Ds*Red colocalization in individual Z-axis slices. In these images; top, GFP signal; middle, *Ds*Red signal; bottom, merge. Central image shows combination of all Z-axis images for the merged GFP and *Ds*Red signals.

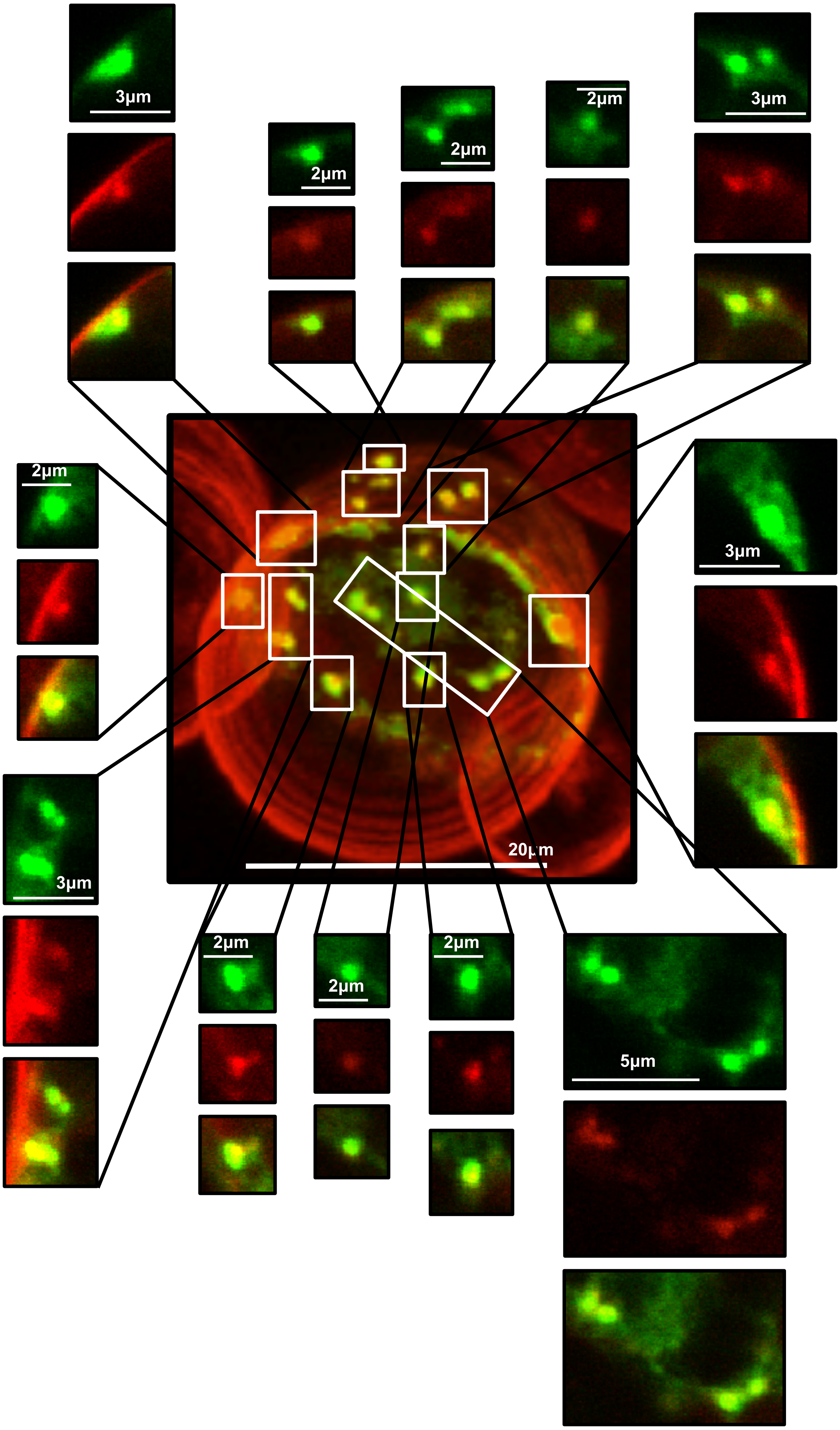

Figure S4. GFP-Adi3/FM4-64 colocalization after wortmannin treatment. Images around central image are confocal microscopy close-up images of GFP/FM4-64 colocalization in individual Z-axis slices. In these images; top, GFP signal; middle, FM4-64 signal; bottom, merge. Central image shows combination of all Z-axis images for the merged GFP and FM4-64 signals.

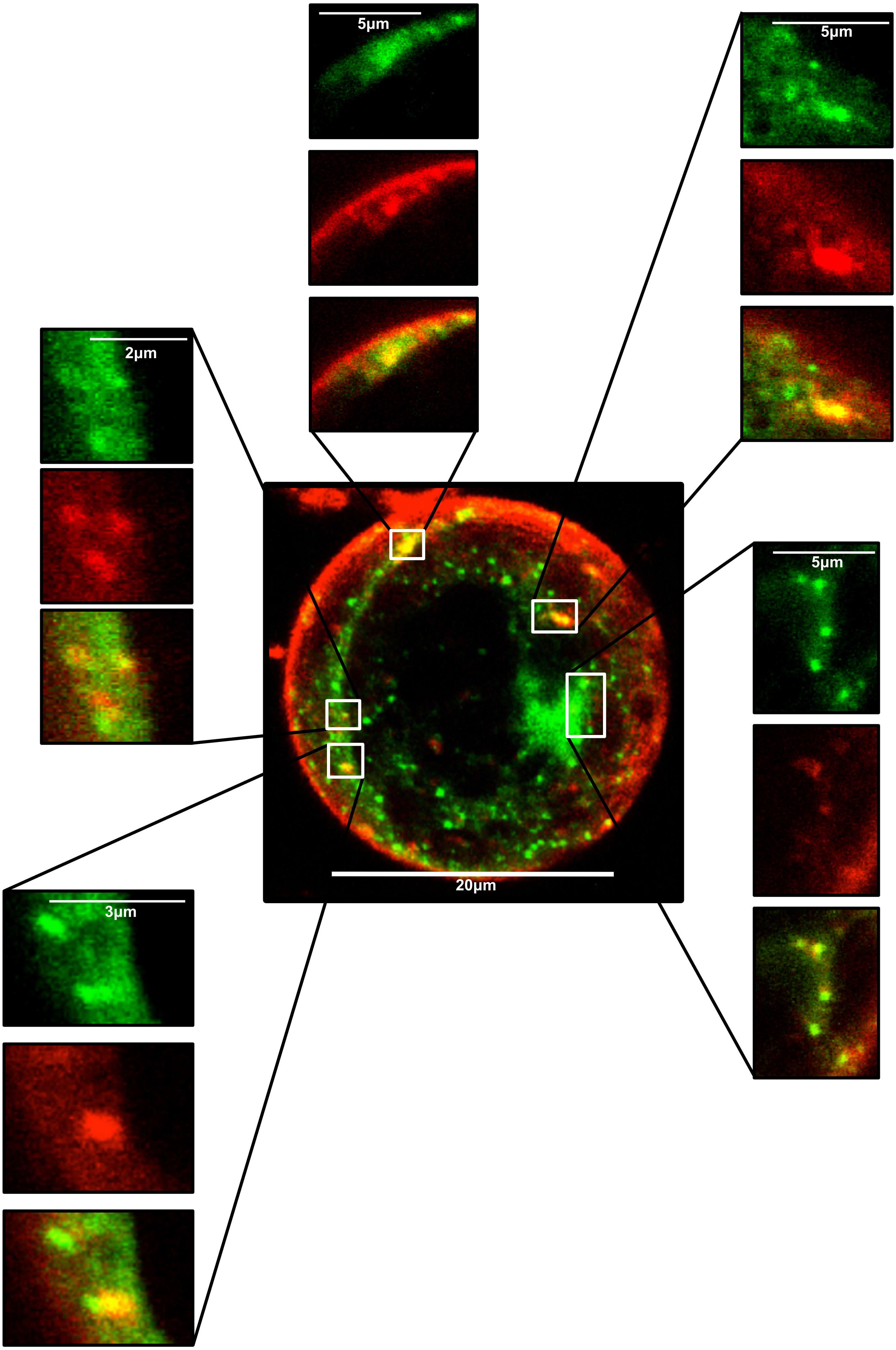

Figure S5. GFP-Adi3/FM4-64 colocalization after brefeldin A treatment. Images around central image are confocal microscopy close-up images of GFP/FM4-64 colocalization in individual Z-axis slices. In these images; top, GFP signal; middle, FM4-64 signal; bottom, merge. Central image shows combination of all Z-axis images for the merged GFP and FM4-64 signals.

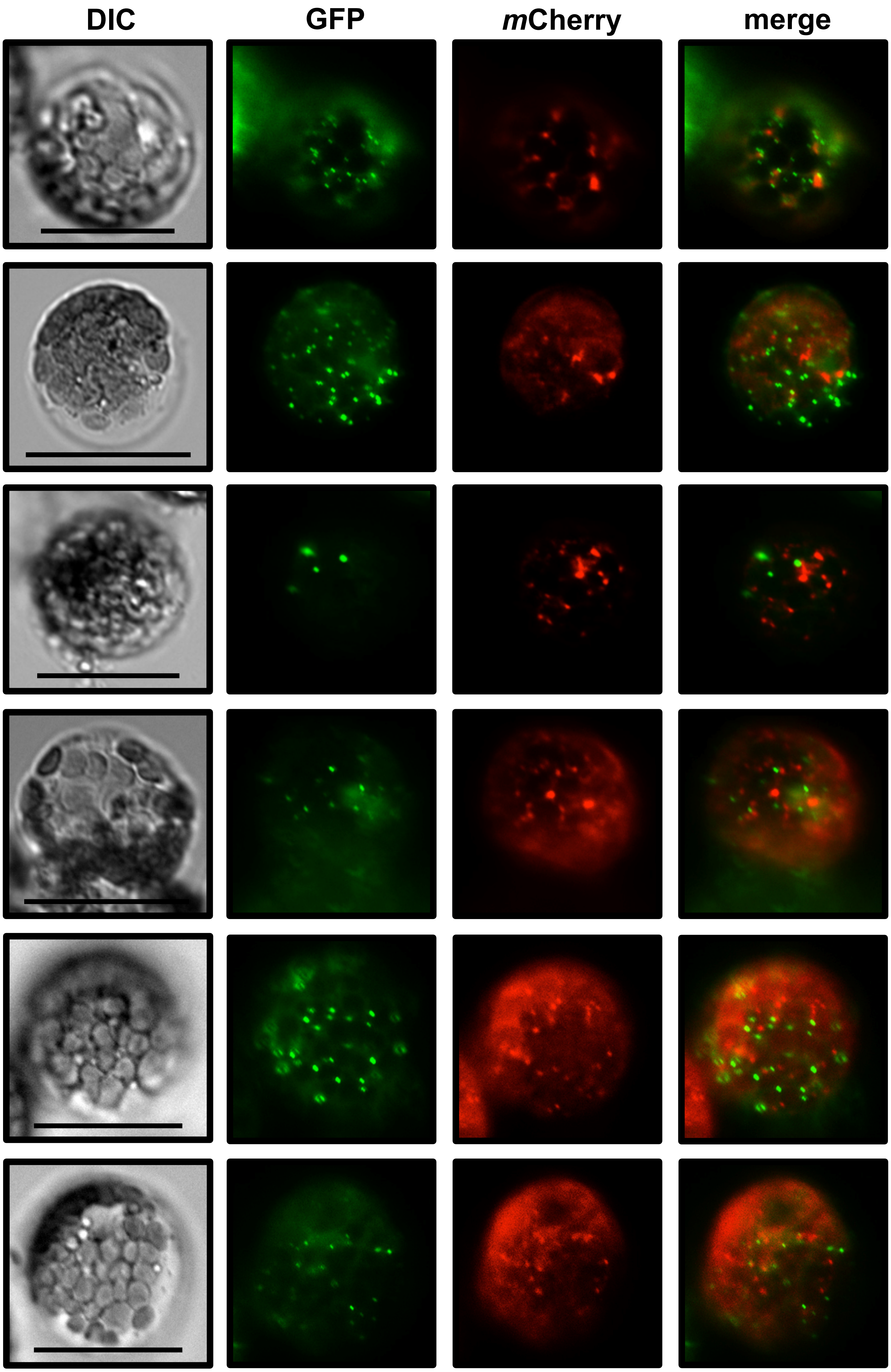

Figure S6. GFP-Adi3$^{\Delta T\text{-loop}}$/*m*Cherry-SYP61 colocalization. Fluorescent microscope images of six independent protoplasts showing localization of GFP-Adi3$^{\Delta T\text{-loop}}$ and *m*Cherry-SYP21. Constructs were cotransformed into protoplasts, incubated for 16 hrs, and 50 µM dexamethasone added to induce *m*Cherry-SYP21 expression. Cells were viewed by fluorescent microscopy XX hrs after dexamethasone treatment. The *m*Cherry-SYP21 construct was described previously (Gu and Innes, 2011, Plant Physiol. **155**:1827). Bar = 20 µm.

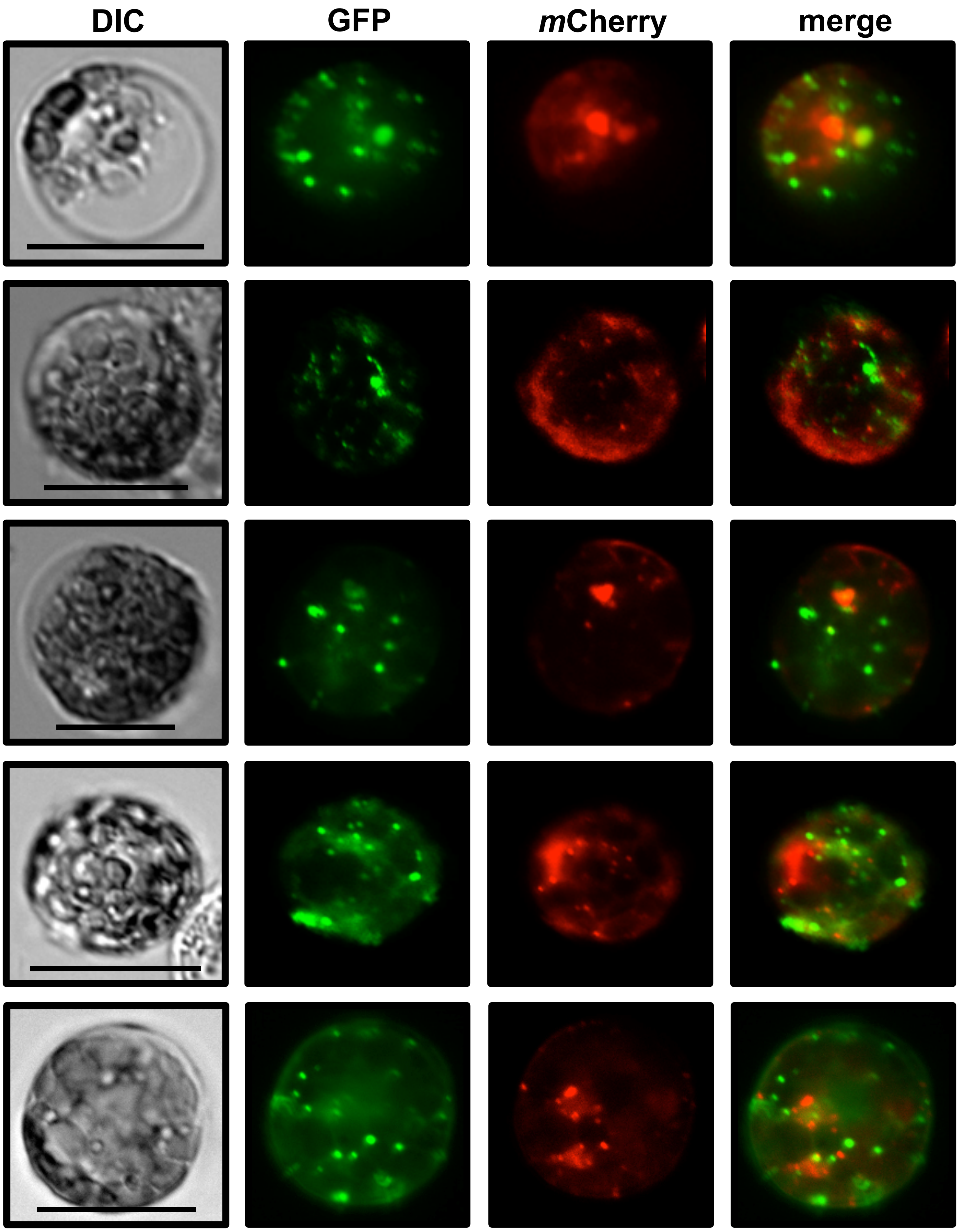

Figure S7. GFP-Adi3$^{\Delta T\text{-loop}}$/*m*Cherry-SYP21 colocalization. Fluorescent microscope images of five independent protoplasts showing localization of GFP-Adi3$^{\Delta T\text{-loop}}$ and *m*Cherry-SYP21. Constructs were cotransformed into protoplasts, incubated for 16 hrs, and 50 μM dexamethasone added to induce *m*Cherry-SYP21 expression. Cells were viewed by fluorescent microscopy XX hrs after dexamethasone treatment. The *m*Cherry-SYP21 construct was described previously (Gu and Innes, 2011, Plant Physiol. **155**:1827). Bar = 20 μm.

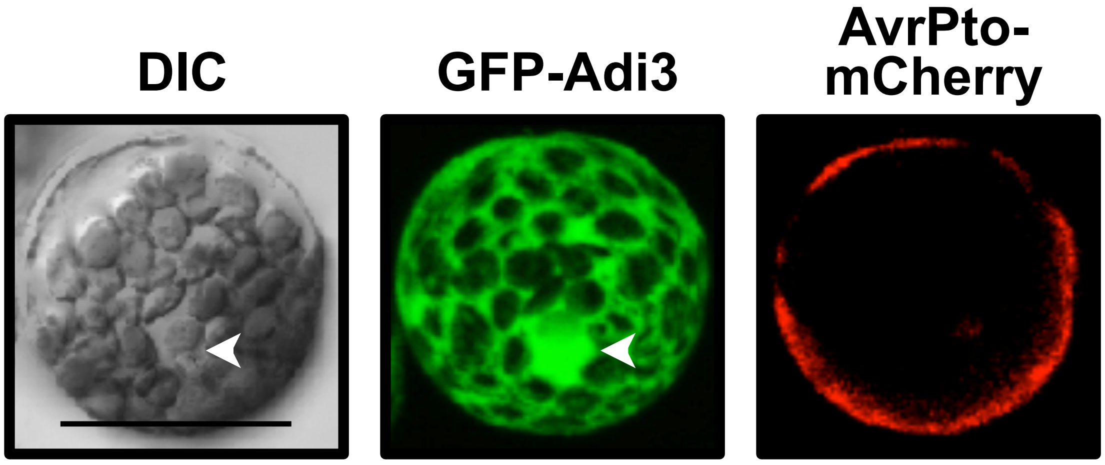

Figure S8. GFP-Adi3 localization in the presence of AvrPto in *prf-3* tomato protoplasts. *prf-3* protoplasts expressing GFP-Adi3 for 16 hrs were transformed with an *AvrPto-mCherry* construct, the protein allowed to express for an additional 4 hrs, and the GFP and mCherry signals viewed by confocal microscopy. GFP and mCherry were viewed in the same cell. Arrowhead, nucleus; bar, 20 μm
